# Determinants of health facility delivery among young mothers aged 15 – 24 years in Nigeria: a multilevel analysis of the 2018 Nigeria demographic and health survey

**DOI:** 10.1186/s12884-023-05492-x

**Published:** 2023-03-17

**Authors:** Tope Olubodun, Semiu Adebayo Rahman, Oluwakemi Ololade Odukoya, Ifeoma P. Okafor, Mobolanle Rasheedat Balogun

**Affiliations:** 1 Department of Community Medicine and Primary Care, Federal Medical Center, Abeokuta, Ogun State Nigeria; 2 Department of Demography and Social Statistics, Obafemi Awolowo University, Ile Ife, Osun State Nigeria; 3 Department of Community Health and Primary Care, College of Medicine, University of Lagos, Lagos, Nigeria

**Keywords:** Determinants, Health facility delivery, Institutional delivery, Skilled birth attendance, Young Mothers, Community, Nigeria

## Abstract

**Background:**

Young mothers aged 15 to 24 years are particularly at higher risk of adverse health outcomes during childbirth. Delivery in health facilities by skilled birth attendants can help reduce this risk and lower maternal and perinatal morbidity and mortality. This study assessed the determinants of health facility delivery among young Nigerian women.

**Methods:**

A nationally representative population data extracted from the 2018 Nigeria Demographic and Health Survey of 5,399 young women aged 15–24 years who had had their last birth in the five years before the survey was analysed. Data was described using frequencies and proportions. Bivariate and multivariate analyses were carried out using Chi-Square test and multilevel mixed effect binary logistic regression. All the analysis were carried out using STATA software, version 16.0 SE (Stata Corporation, TX, USA)..

**Results:**

Of the total sampled women in the 2018 NDHS, 5,399 (12.91%) formed our study population of young women 15 -24 years who had their last birth in the preceding five years of the survey. Only 33.72% of the young mothers utilized health facility for delivery. Women educated beyond the secondary school level had 4.4 times higher odds of delivering at a health facility compared with women with no education (AOR 4.42 95%, CI 1.83 – 10.68). Having fewer children and attending more antenatal visits increased the odds of health facility delivery. With increasing household wealth index, women were more likely to deliver in a health facility. The odds of health facility delivery were higher among women whose partners had higher than secondary level of education. Women who lived in communities with higher levels of female education, skilled prenatal support, and higher levels of transportation support were more likely to deliver their babies in a health facility.

**Conclusion:**

Strategies to promote institutional delivery among young mothers should include promoting girl child education, reducing financial barriers in access to healthcare, promoting antenatal care, and improving skilled birth attendants and transportation support in disadvantaged communities.

## Introduction

Globally, approximately 810 women die from complications of pregnancy or childbirth each day [1] For each woman who dies, approximately 20 others suffer serious infections, injuries or disabilities [2] Sub-Saharan Africa has the highest maternal mortality ratio of 533 maternal deaths per 100,000 live births, which corresponds to 200,000 maternal deaths a year [2] and nearly 20% of all global maternal deaths happen in Nigeria [3]. Nigeria’s estimated maternal mortality ratio is 512 maternal deaths per 100,000 live births according to NDHS 2018 [4] and the lifetime risk of dying during pregnancy, childbirth or postpartum/post-abortion for a Nigerian woman is 1 in 33 [4].

Maternal mortality is considered an individual tragedy and a human rights violation as most times, they are preventable [5]. The impact of maternal mortality and morbidity can be far reaching affecting families and communities. Maternal mortality can have adverse health and psychological effects for children, the spouse and other household members [6]. There is a link between maternal mortality/morbidity and increased risk of stillbirth and neonatal deaths [7, 8]. Surviving older children may suffer from disruptions in education and also living arrangements, leaving these children as victims to the cycle of poverty, who are thus at higher risks of repeating maternal and neonatal mortality [9–11]. Surviving children may also be more vulnerable to illnesses and malnutrition [11]. Spouses are often bereft, and ill-prepared to handle the role expansion required after losing a wife [12]. Economic losses and poverty often follows maternal death, as many times, additional income is lost, and there are economic costs to the family associated with illness and death [12]. Communities and even societal norms and behaviors may be affected by illness or death, especially if the sick or deceased woman is or was a prominent member of the community [6].

Maternal mortality shows elevated rates at extremes of maternal age [13]. Young mothers are particularly at higher risk of adverse health outcomes. Teenage pregnancies have major health consequences for the mothers and their babies and these include higher risks of hypertensive disorders of pregnancy, gestational diabetes, anemia in pregnancy, delivery complications, puerperal endometritis and systemic infections [14, 15]. Babies born to adolescents also face higher risks of preterm delivery, low birth weight, birth trauma, respiratory diseases and severe neonatal conditions [14, 15]. Compared to older mothers, young mothers are at higher risks of having unplanned pregnancies and sexually transmitted infections (including HIV) and tend to have lower educational attainment, lower earnings, and poorer health [16–18]. These vulnerabilities can impact on delivery outcomes.

The process of childbirth can result in unexpected complications [19]. Three quarters of maternal deaths occur during delivery and in the immediate post-partum period [19]. Health facility delivery provides skilled health attendants to better manage the outcome of pregnancy and child birth and has a positive contribution in reducing maternal and newborn mortality and morbidity. The link between early and regular antenatal care attendance, delivery in health facility, and improved maternal health outcomes has been well documented for a considerable amount of time [20]. Women who deliver in health facilities have access to basic obstetric care, neonatal care, and emergency care, hence improved, maternal and neonatal health outcomes [19].

In Nigeria, efforts to improve health facility delivery led to the introduction of the Midwives Service Scheme and the Subsidy Reinvestment and Empowerment Programme (SURE-P) [21, 22]. The Nigerian government in 2009 set up the Midwives service scheme to improve availability of skilled birth attendants in rural areas in the country in a bid to increase health facility delivery and quality of healthcare [21]. The program engages newly graduated, unemployed and retired midwives to work temporarily in rural areas. In addition, the SURE-P was initiated in 2012 to re-invest fuel subsidy funds into social safety net programs which included improving maternal health. The SURE – P had a component of conditional cash transfer to women for attending four antenatal care visits, delivering in a health facility and attending postnatal visits [21, 22]. Other components of the SURE – P include health facility staffing and renovations, supply chain for essential maternal health commodities, and community mobilization through village health workers and leadership committees [22]. These programmes recorded some successes, however, in 2018, only about 33% of young women in Nigeria delivered at a health facility [23].

Identifying factors associated with health facility delivery among young women is pertinent to providing information for interventions and policies aimed at reducing maternal mortality. Current literature does not adequately address this gap. For instance, Ononokpono et. al, [24]. Adedokun et. al, [25] and Solanke et. al. [26] have identified determinants of place of delivery among Nigerian women aged 15–49 years without specific attention to young mothers who have the highest risk. This study however, will focus on young women as they form an important high risk group for maternal mortality and morbidity. Olakunde et. al. [27] and Rai et. al. [28] examined factors associated with skilled birth attendants at delivery among married adolescent girls in Nigeria, however the factors examined were limited to individual characteristics such as educational attainment, wealth quintile, pregnancy wantedness, parity and antenatal care (ANC) visit [27]. Community characteristics also influence place of delivery to a large extent and this information can inform strategies to reduce maternal mortality. Our study considers the hierarchical structure of the Nigerian Demographic and Health Survey (NDHS) data and thus employs a multilevel modeling to assesses both individual and community levels characteristics. Insight into the community attributes impacting health facility delivery is essential for programs, policies and strategies aimed at increasing health facility delivery among young mothers as efforts can be best directed at communities with the most need. This study assessed the factors associated with health facility delivery among young women aged 15 to 24 years in Nigeria using data from the most recent Demographic and Health Survey.

## Methods

### Data source

Women recode data extracted from the Nigerian Demographic and Health Survey 2018 was analysed. The 2018 NDHS is the sixth Demographic and Health Survey conducted in Nigeria since 1990 [29]. Data collection took place from 14 August 2018 to 29 December 2018 [4]. The survey was cross-sectional and provides estimates of demographic and health indicators [29].

### Sampling methodology of the 2018 NDHS

The Population and Housing Census of the Federal Republic of Nigeria (NPHC), conducted in 2006 was the sampling frame used for the 2018 NDHS [29]. The primary sampling unit (PSU)/cluster for the 2018 NDHS is defined on the basis of enumeration areas (EAs) from the 2006 census. A nationally representative sample of respondents were interviewed in the 6 geographical zones, 36 states and the Federal Capital Territory (FCT) [29]. Stratified sampling in two stages was used to select respondents [4]. The 37 states were separated into urban and rural areas such that in total, there were 74 sampling strata. In the first stage, 1,400 EAs were selected with probability proportional to EA size. In the second stage, 30 households were selected in each cluster by an equal probability systematic sampling. A sample of 41,821 women aged 15–49 in 40,427 households participated in the survey. This study is however limited to 5,399 women aged 15 – 24 years who had recent live birth in the preceding five years of the survey.

### Study variables

The dependent and independent variables examined in this study with their descriptions are presented in Table 1.

**Table 1:** Description of study variables

Study variables	Description	Categorizations
**Outcome variable**		
Health facility delivery	A health facility delivery was when the most recent childbirth took place in a government hospital, government health center, government health post, other public sector, private hospital/clinic or other private facility When a delivery took place in a respondent’s home, other home, or other places, it was not a health facility delivery	▪ Utilized health facility for delivery ▪ Did not utilize health facility for delivery
**Individual Level Variables**		
Age at childbirth	Age of the mother when she had the most recent childbirth	▪ 15 – 19 years ▪ 20 – 24 years
Marital status	Women not married were defined as those never in union and those that were formerly in union/living with a man Married women were defined as women currently in union/living with a man	▪ Not married ▪ Married
Highest level of education	The highest level of formal education attained	▪ No education ▪ Primary ▪ Secondary ▪ Higher
Employment status	If the respondent was employed at time of survey	▪ currently working ▪ Not currently working
Wanted index pregnancy	Refers to if the respondent wanted the pregnancy when she became pregnant	▪ Pregnancy wanted ▪ Pregnancy not wanted
Number of childbirths	The number of children ever born to the respondent	▪ 1 ▪ 2–3 ▪ 4–7
Number of antenatal visits	Number of antenatal visits in the index pregnancy	▪ No ANC visits ▪ less than four visits ▪ at least four visits
Distance to health facility	Refers to how much of a problem is ‘the distance to the health facility’ in getting medical help for oneself	▪ A big problem ▪ Not a big problem
Household wealth index	Household wealth index in the NDHS is divided into five equal categories; poorest, poorer, middle, richer, richest In this study, we recoded wealth index into 3 categories with ‘poor’ comprising of poorest and poorer, ‘middle’ comprising of middle and ‘rich’ comprising of richer and richest	▪ Poor ▪ Middle ▪ Rich
Exposure to mass media	Mass media exposure was generated from exposure to television, radio and newspaper Mass media exposure was defined as ‘exposed’ for those with access to at least one of television, radio or newspaper, and ‘no exposure’ for those who had no access to any of these	▪ No exposure ▪ Exposed
Participates in healthcare decision	This refers to whether respondent participates in decision on her healthcare This variable was derived from the variable—person who usually decides on respondent’s health care A respondent participates if the decision is made by respondent alone, or respondent and partner A respondent does not participate when the decision is made by her partner alone, or someone else	▪ Participates: ▪ Does not participate
Partner’s level of education	The highest level of education of respondent’s partner	▪ No education ▪ Primary ▪ Secondary ▪ Higher
Partner’s employment status	This refers to whether the partner was employed at the time of the survey or not	▪ Currently employed ▪ Not currently employed
Community Variables		
Community level poverty	Community level poverty was defined as the proportion of women who are from the poorest and poorer communities	▪ Low ▪ Medium ▪ High
Community level women’s education	Community level women’s education was defined as proportion of women from community with at least secondary education	▪ Low ▪ Medium ▪ High
Community level of skilled prenatal support	Community skilled prenatal care was defined as proportion of women from community with availability of antenatal care from a skilled health provider (doctor, nurse, midwives)	▪ Low ▪ Medium ▪ High
Community level of transportation support	Community level of transportation was defined as the proportion of women from community with viable means of transportation to health facility	▪ Low ▪ Medium ▪ High
Ethnic diversity	Ethnic diversity refers to the concentration of different ethnic groups in a community It was defined as the proportion of women from different ethnic groups in the primary sampling unit The value ranges from 0 to 100. A value of 0 (low) reflects a mono-ethnic community, whereas a value of 100 (high) relects that the community is multi-ethnic in nature	▪ Low ▪ Medium ▪ High
Place of residence	The place of residence if urban or rural	▪ Urban ▪ Rural
Region	This is the geographical region which the respondent lives	▪ Northcentral ▪ Northeast ▪ Northwest ▪ Southeast, ▪ South-south ▪ Southwest

Community level poverty, community level women’s education, community level of skilled prenatal support, community level of transportation support and ethnic diversity were computed by aggregating individual characteristics at the cluster level (primary sampling unit), dividing the measure into tertiles and categorizing as low, medium and high. Similar procedure has been widely applied to derive community variables in DHS datasets [21–23]

#### Data analysis

Weighted data analysis was done using STATA software, version 16.0 SE (Stata Corporation, TX, USA). Three levels of analysis were carried out. First, descriptive analysis was done to determine the distribution of respondents in terms of individual characteristics and community levels characteristics. Second, bivariate analysis was done to determine the association between the given characteristics and place of delivery using Chi-square to test the statistical significance. Third, multilevel logistic regression analysis was used to account for the hierarchical nature of the DHS data. We estimated four models. The first model being an empty model, contained no covariates but decomposed the total variance into individual and community components. The second model included individual characteristics only. The third model included only the community level variables, while the fourth model included both the individual and community levels variables.

Odds ratios were used to present the results of fixed effect in addition with the confidence interval (95%). Intra cluster correlation (ICC) was used to explain the results of random effect. Model goodness of fit was checked using BIC, multi-collinearity was confirmed through application of Variance Inflation Factor (VIF) and the variable – marital status—was dropped from the regression analysis due to multi-collinearity. The mathematical statement of the multilevel mixed effect binary logistic regression model is as follows:

Empty Model (Model 0): The model expresses the similarity in the health facility delivery among young mothers across the communities.

$$Log\left[\frac{{\pi }_{ij}}{1-{\pi }_{ij}}\right]={\beta }_{0ijk}+{e}_{ij}$$

Other models that contain explanatory variables:

$$Log\left[\frac{{\pi }_{ij}}{1-{\pi }_{ij}}\right]={\beta }_{0}+{\beta }_{1}{X}_{1ij}+....{\beta }_{n}{X}_{nij}+{u}_{oj}+{e}_{ij}$$

Where:*πij* is the log of odds of delivery outside of health facility

*(1-πij*) is the log of odds of health facility delivery*β0* is log odds of the intercept*β1, … βn* are changes in level of health facility delivery due to individual and community-level factors

*X1ij… Xnij* are independent variables of individuals and communities

*U0*j are random errors at community levelseij is the error term or residuals

#### Ethical approval

Being a secondary data, we registered and obtained permission to download the requested datasets from the measure DHS website. The data were handled with confidentiality. The 2018 NDHS survey protocol was approved by the National Health Research Ethics Committee of Nigeria (NHREC) and the ICF Institutional Review Board. Written informed consents were obtained from all participants. **A**ll methods were performed in accordance with the Declaration of Helsinki.

## Results

Of the 5,399 young mothers, only 33.72% (one third) of the young mothers aged 15–24 years utilized health facility for delivery. About half (49.29%) had their most recent childbirth between the ages of 12 and 19 while the remaining half gave birth in their early twenties (20 -24 years). Majority (91.02%) were currently married/living with spouse/partner. Most of the women had secondary education as their highest level of education (35.74%). Half of the respondents (51.85%) had at least four antenatal visits and 27.98% did not receive antenatal care. A higher proportion (45.62%) of the respondents fell into the poor household wealth index and 45.33% had no exposure to mass media. Majority of the respondents resided in rural communities (70.84%) (Table 2).

**Table 2:** Sample characteristics and prevalence of health facility delivery for mothers aged 15–24 years In Nigeria, 2018 NDHS

Variables	Frequency	Percentage
**Health Facility Delivery**		
Utilize health facility for delivery	1874	33.72
Did not utilize health facility for delivery	3525	66.28
**Age at last childbirth**		
12 – 19	2631	49.29
20 -24	2768	50.71
**Marital Status**		
Not married	554	8.98
Married	4845	91.02
**Highest Level of Education**		
No education	2533	48.50
Primary	743	13.74
Secondary	1981	35.28
Higher	142	2.48
**Employment status**		
Currently working	2546	46.47
Not currently working	2853	53.53
**Number of children born**		
1	2533	46.46
2–3	2519	47.07
4–7	347	6.47
**Wanted Index Pregnancy**		
Pregnancy wanted	4629	87.15
Pregnancy not wanted	1770	2.85
**Number of Antenatal visits**		
No ANC	1517	27.98
Less than 4	1088	20.17
At least 4	2738	51.85
**Getting medical help for self: Distance to Health Facility**		
A big problem	1787	30.78
Not a big problem	3612	69.22
**Wealth Index**		
Poor	2509	45.62
Middle	1214	23.56
Rich	1676	30.82
**Exposure to Mass media**		
No exposure	2489	45.33
Exposed	2910	54.67
**Participates in Healthcare Decision**		
Participates	1458	29.52
Does not Participate	3387	70.48
**Partner’s level of education**		
No education	1931	41.73
Primary	594	12.83
Secondary	1724	34.48
Higher	524	10.96
**Partner’s employment status**		
Not currently employed	180	3.78
Currently employed	4651	96.21
**COMMUNITY VARIABLES**		
**Community Poverty**		
Low	1230	21.48
Medium	1788	36.23
High	2381	42.29
**Community women’s education**		
Low	2895	55.74
Medium	1561	27.65
High	943	16.61
**Community skilled prenatal support**		
Low	1998	35.11
Medium	1494	27.72
High	1907	37.17
**Community transportation support**		
Low	2339	41.68
Medium	1788	33.53
High	1272	24.79
**Ethnic diversity**		
Low	1776	30.81
Medium	1921	39.38
High	1702	29.81
**Place of residence**		
Urban	1424	29.16
Rural	3975	70.84
**Region**		
Northcentral	932	13.93
Northeast	1348	20.96
Northwest	1847	42.34
Southeast	375	6.268
South-south	469	7.222
Southwest	428	9.284

Ethnic diversity is the proportion of women from different ethnic groups in the primary sampling unit

The results of the bivariate analysis indicate that all the independent variables except ‘partner’s employment status’ and ‘ethnic diversity’ were significantly associated with health facility delivery. A higher proportion of those that were currently married/living with partner (48.67%) utilized health facility for delivery. Utilization of health facility delivery was highest for those who had post-secondary school education (80.76%) compared with women with secondary education (56.01%), primary education (34.25%) and no education (14.94%). Delivery in a health facility was highest for women who had only one childbirth (42.07%) compared with those with 2–3 childbirths (26.94%) and those with 4 -7 childbirths (23.13%). A higher proportion of women who had at least 4 antenatal visits (50.00%) utilised health facility for delivery, compared with those who had less than 4 visits (28.30%) and those who didn’t attend antenatal care (5.50%) (Table 3).

**Table 3:** Bivariate analysis of factors associated with health faciity delivery among young mothers aged 15–24 Years In Nigeria

VARIABLE	Proportion that Utilized Health Facility Delivery (%)	Chi Square	*P* value
**Age at last childbirth**			
12 – 19	29.44	42.9872	< 0.0001
20 -24	37.88		
**Marital Status**			
Not married	48.67	53.2070	< 0.0001
Married	32.25		
**Highest Level of Education**			
No education	14.94	969.4764	< 0.0001
Primary	34.25		
Secondary	56.01		
Higher	80.76		
**Employment status**			
Currently working	40.09	112.8733	< 0.0001
Not currently working	26.38		
**Number of children born**			
1	42.07	148.0455	< 0.0001
2–3	26.94		
4–7	23.13		
**Wanted Index Pregnancy**			
Pregnancy wanted	31.27	98.5057	< 0.0001
Pregnancy not wanted	50.35		
**Number of Antenatal visits**			
No ANC	5.50	881.6583	< 0.0001
Less than 4	28.30		
At least 4	50.00		
**Getting medical help for self: Distance to Health Facility**			
A big problem	26.18	61.1137	< 0.0001
Not a big problem	37.07		
**Wealth Index**			
Poor	23.07	280.9105	< 0.0001
Middle	35.51		
Rich	48.11		
**Exposure to Mass media**			
No exposure	20.2	366.0355	< 0.0001
Exposed	44.93		
**Participates in Healthcare Decision**			
Participates	46.5	188.7203	< 0.0001
Does not Participate	26.28		
**Partner’s level of education**			
No education	12.97	745.7675	< 0.0001
Primary	27.03		
Secondary	49.17		
Higher	60.11		
**Partner’s employment status**			
Not currently employed	25.18	4.4599	0.1941
Currently employed	32.62		
**COMMUNITY VARIABLES**			
**Community Poverty**			
Low	49.09	324.5518	< 0.0001
Medium	39.69		
High	20.79		
**Community women’s education**			
Low	14.98	1128.9759	< 0.0001
Medium	51.51		
High	67.01		
**Community skilled prenatal support**			
Low	17.6	345.4393	< 0.0001
Medium	40.19		
High	44.12		
**Community transportation support**			
Low	19.92	614.0985	< 0.0001
Medium	31.34		
High	60.13		
**Ethnic Diversity**			
Low	35.08	20.3187	0.0537
Medium	30.23		
High	36.92		
**Place of residence**			
Urban	53.67	395.6539	< 0.0001
Rural	25.51		
**Region**			
Northcentral	51.89	1027.9402	< 0.0001
Northeast	27.65		
Northwest	15.76		
Southeast	76.56		
South-south	38.49		
Southwest	69.45		

A higher proportion of young mothers residing in communities with low level of poverty (49.09%) had their most recent delivery in a health facility. Lower proportions of respondents who live in communities with low levels of education (14.98%) utilized health facility delivery. Respondents residing in communities with high level of transportation support had higher utilization of health facility for delivery (60.13%). Women who reside in rural areas (25.51%) had lower utilization of facility delivery than women residing in urban areas. Women residing in southeast Nigeria (76.56%), the had highest utilization of facility delivery while women residing in Northwest Nigeria (15.76%) had the lowest utilization of facility delivery (Table 3).

Table 4 shows the multivariate result. The parameters of the model such as the AIC and BIC confirmed that the models were well fitted The Log likelihood further reflected the statistical significance of random effects. As shown in the Table 4, when no independent factors were included in the analysis (empty model), the proportion of variation in the health facility delivery was 66.54% between communities. This therefore indicated significant variation in the health facility delivery among young mothers across the communities. This finding suggests that some communities deliver at health facility than others. Model 2 which contains only the individual level variables show that highest level of education, employment status, number of children born, number of antenatal visits, wealth index, exposure to mass media, partner’s level of education were significantly associated with health facility delivery. The results of ICC reflected reduction in the variation of health facility delivery among youngers to 49.56%. Model 3 contains only the community level variables and reveals that community level of poverty, community women’s education, community skilled prenatal support, and community transportation support were significantly associated with health facility delivery. The results of ICC indicated further reduction in the variation in health facility delivery to 47.18% when only community variables when fitted into the model. At model 4 however, exposure to mass media and community level of poverty were not predictors of health facility delivery. From the 4^th^ model (model consisting of individual and community explanatory variables), women with higher than secondary level of education had 4.4 times higher odds of delivering at a health facility than women with no education. Women with secondary school education had 1.5 times higher odds of delivering in a health facility, compared to women with no education. The more the number of children born to a woman, the lower the odds of delivering in a health facility and the more antenatal visits a woman attends, the more likely she will deliver in a health facility. The respondents that fell into the middle (AOR 1.52; 95% CI 1.11—2.08) rich (AOR 1.67;95% CI 1.17—2.39) household wealth index were more likely to deliver in a health facility than those that fell into the poor household wealth index. The odds of health facility delivery were higher among women whose partners had higher than secondary education (AOR 1.69 95%; CI 1.08—2.64) compared to those whose partners had no education (Table 4).

**Table 4:** Multilevel analysis showing determinants of health faciity delivery among young mothers aged 15–24 years In Nigeria

VARIABLE	Model 1	Model 2	Model 3	Model 4
	**Empty Model**	**Individual variables**	**Community variables**	**Individual/Community variables**
		**Adjusted Odds ratio**	**Adjusted Odds ratio**	**Adjusted Odds ratio**
**Age at last childbirth**				
12 – 19		1		1
20 -24		1.19 (0.94—1.50)		1.08 (0.86—1.36)
**Highest Level of Education**				
No education		1		1
Primary		1.78 (1.24—2.54)**		1.25 (0.91 – 1.36)
Secondary		3.07 (2.01- 4.67)***		1.53 (1.09—2.14)*
Higher		12.93 (4.55—36.76)***		4.42 (1.83 – 10.68)**
**Employment status**				
Currently working		1.43 (1.11—1.83)**		1.23 (0.98 – 1.55)
Not currently working		1		1
**Number of children born**				
1		1		1
2–3		0.39 (0.28—0.55)***		0.43 (0.32—0.58)***
4–7		0.42 (0.24—0 .71)**		0.44 (0.26—0.73)**
**Wanted Index Pregnancy**				
Pregnancy wanted		0.97 (0.68—1.38)		0.97 (0.68 – 1.37)
Pregnancy not wanted		1		1
**Number of Antenatal visits**				
No ANC		1		1
Less than 4		8.36 (4.42—15.78)***		6.81 (3.90 – 11.94)***
At least 4		20.00(8.97—44.61)***		13.49 (6.89—26.42)***
**Getting medical help for self: Distance to Health Facility**				
A big problem		1		1
Not a big problem		1.16 (0.89—1.50)		1.21 (0.93—1.56)
**Wealth Index**				
Poor		1		1
Middle		1.57 (1.15—2.15)**		1.52 (1.11—2.08)**
Rich		1.69 (1.22—2.32)**		1.67 (1.17—2.39)**
**Exposure to Mass media**				
No exposure		1		1
Exposed		1.46 (1.12—1.90)**		1.22 (0.96—1.57)
**Participates in Healthcare Decision**				
Participates		1		1
Does not Participate		1.25 (0.97—1.61)		0.98 (0.77—1.23)
**Partner’s level of education**				
No education		1		1
Primary		1.30 (0.89—1.89)		0.87 (0.60—1.25)
Secondary		2.00 (1.38—2.90)***		1.18 (0.86—1.62)
Higher		2.59 (1.56—4.31)***		1.69 (1.08—2.64)*
**COMMUNITY VARIABLES**				
**Community level of Poverty**				
Low			1	1
Medium			0.98 (0.71—1.35)	1.18 (0.84—1.66)
High			0.65 (0.44—0.96)*	1.37 (0.88 – 2.14)
**Community women’s education**				
Low			1	1
Medium			4.01 (2.62—6.14)***	2.09 (1.41 – 3.10)***
High			6.48 (3.58—11.73)***	2.49 (1.42 – 4.38)**
**Community skilled prenatal support**				
Low			1	1
Medium			2.11 (1.51—2.96)***	1.50 (1.41 – 3.10)***
High			3.21 (2.21—4.67)***	1.73 (1.42—4.38)**
**Community transportation support**				
Low			1	1
			1.94 (1.42—2.65)***	1.69 (1.24—2.31)*
High			4.61 (2.93—7.26)***	3.75 (2.34—5.99)*
**Place of residence**				
Urban			1	1
Rural			0.99 (0.71—1.36)	0.16 (0.82—1.63)
**Region**				
Northcentral			1	1
Northeast			0.32 (0.21 – 0.49)***	0.26 (0.16 – 0.42)***
Northwest			0.12 (0.07 – 0.21)***	0.10 (0.53 – 0.20)***
Southeast			1.93 (1.16 – 3.22)*	1.21 (0.72 – 2.06)
South-south			0.14 (0.08 – 0.26)***	0.10 (0.05 – 0.23)***
Southwest			1.06 (0.67 – 1.67)	0.71 (0.44 – 1.16)
**Variance**	1.063(0.230 – 4.870)***	1.542 (0.340—6.995)***	1.9593 (0.7300—5.2587)***	1.366 (0.283- 6.592)***
**ICC(%)**	66.52	49.56	47.18	37.09
**Log Likelihood**	-3001.64	-2109.56	-2541.67	-1913.00
**Model fit Statistics**				
**AIC**	6009.29	4261.11	5117.33	3896.00
**BIC**	6029.07	4396.79	5229.43	4122.13

^*^p < 0.05

^**^p < 0.01

^***^ *p* < 0.001

Women who lived in communities with high (AOR 2.09; 95% CI 1.41 – 3.10) and medium (AOR 2.49; 95% CI 1.42 – 4.38) levels of female education were more likely to have health facility delivery compared with those who reside in communities with low levels of female education. Women who lived in communities with high (AOR 1.50; 95% CI 1.41—3.10) and medium (AOR 1.73; 95% CI 1.42 – 4.38) levels of skilled prenatal support were more likely to have health facility delivery compared with those who reside in communities with low levels of skilled prenatal support. The odds of health facility delivery were higher among women who lived in communities with high (AOR 1.69; 95% CI 1.24 – 2.31) and medium (AOR 3.75; 95% CI 2.34 – 5.99) levels of transportation support compared with those with low transportation support. Women residing in Northeast (AOR 0.26; 95% CI 0.16 – 0.42), Northwest (AOR 0.10; 95% CI 0.53 – 0.20) and South-south (AOR 0.10; 95% CI 0.05 – 0.23) regions were less likely to utilize health facility for delivery compared with women from Northcentral. The value of ICC reduced to 37.09% when individual and community levels variables were fitted into the model. This implies that other factors have impact on health facility delivery apart from the community where the young mothers reside (Table 4).

## Discussion

Only one in three of the young women delivered in health facility. This study showed that women with higher levels of education, women who had fewer children, women who attended more antenatal visits, women with higher wealth index, women whose partners had higher than secondary education were more likely to use health facility for delivery. Young women who lived in communities with higher levels of female education, skilled prenatal support, higher levels of transportation support were more likely to deliver their babies in a health facility.

The association of increased use of health facility for delivery with higher maternal education is similar to other studies that analyzed DHS reports of African countries [30–34]. Low maternal education has been a major impediment toward accessing skilled care at delivery among developing countries [32–37]. Improved health literacy among educated women makes them better informed about health care issues. This will reflect in their healthcare decisions. Partners’ education may have a similar effect as seen from findings in this study, as this will influence the partners’ decisions in issues of the family’s health. Improving education attainment of young women and girls can help improve health literacy, health seeking behaviours and ultimately, health facility delivery. Although Nigeria has made basic education officially free and compulsory, about 10 million of the country’s children aged 5–14 years are not in school [38]. Also more than just basic education would be required to influence young women’s attitudes and health seeking behaviours.

As number of children increased, the likelihood to have a facility delivery decreased. This was also reported among youth in Uganda [39] and among adolescents in Bangladesh [35]. This finding may be due to attitudinal factors as women who have more children may feel that they are more experienced with childbirth or because having a large family size means fewer resources to seek skilled delivery care. Another explanation could be that if the women had experienced poor quality care, or disrespect by health workers in a previous health facility delivery, they may choose not to have another health facility delivery. This calls for the need for proper training and supervision of health workers to ensure respectful maternal care.

Household wealth index was used as a proxy to socioeconomic status. With higher household wealth index, women were more likely to deliver at a health facility. Cost of services to deliver at a health facility is usually higher than at unorthodox centres or at home, hence this relationship [40]. Women who are poor may also find it difficult to afford transportation costs to health facilities compared with wealthier women. To reduce inequities between the rich and the poor, reducing financial barriers in access to health facility delivery is critical. This can be achieved by a functional health insurance scheme or free health schemes for poor pregnant women.

Having antenatal care of ≥ 4 visits and 1 – 3 visits had higher odds of facility delivery when compared to having no ANC visits. Women with ≥ 4 visits were 13 times more likely to utilize health facility for delivery. This finding is consistent with previous studies [34, 36, 37, 41]. Knowledge about the benefits of having skilled delivery care is likely to be higher among women who have regular antenatal visits [24]. Women who have regular antenatal visits are more likely to be knowledgeable about the consequences of complicated pregnancy, as well as the risks associated with home delivery. In addition, the same factors that influence utilization of antenatal care such as higher level of education, higher socioeconomic status may also influence choice of place of delivery.

Adolescents and young women often have lower levels of educational attainment and lower earnings and many don’t possess decision making autonomy. In our study less than a third of the participants participated in healthcare decisions, and our findings show that participation in healthcare decision did not influence the odds of health facility delivery. In a study among women of reproductive age using the 2018 NDHS however, high community level of female autonomy (proportion who solely takes decision or jointly with male partner on own healthcare) was associated with higher odds of institutional delivery [26]. This may imply that even when young women participate in decision making, they may not be empowered to make the right decisions regarding their health.

Identifying community variables that influence place of delivery can help inform maternal mortality reduction strategies. Solanke et al., reported that community skilled prenatal support and community transport support were associated with health facility delivery among women 15 to 49 years in Nigeria [26]. Our study found that the community characteristics: community level of poverty, community women’s education, community skilled prenatal support and community transport support influenced place of delivery. In designing interventions, special attention needs to be paid to communities with lower education, and higher levels of poverty. Schemes that provide skilled prenatal support for women in form of provision and equipping of skilled birth attendants in disadvantaged communities should be encouraged.

Young women living in communities with better transportation support were more likely to utilize health facility for delivery. Adolescents and young women may be less empowered to overcome obstacles including transportation obstacles in seeking healthcare. They may not have enough will power and disposable income to pay for more viable means of transportation, hence the association observed in our study. Interventions aimed at improving transportation support for women in labor including community led projects, and government led efforts should be instituted to encourage young women to visit health facilities which are often times a reasonable distance from their homes.

Women in Northeast and Northwestern regions of the country were less likely to utilize health facility for delivery. This finding is consistent with a secondary analysis of the 2008 NDHS [24]. Factors responsible for this may include the high level of poverty, illiteracy and also sociocultural beliefs in these regions [42, 43]. Women from the South-south region were also less likely to deliver in health facilities. The distribution of health facilities being fewer in the Northeast, Northwest and South-south zones could also contribute to the lower prevalence of health facility delivery in these regions [44].

### Limitations

This study is not without limitations. The 2018 NDHS data were collected retrospectively and may be associated with recall bias. Due to the cross-sectional nature of the survey, it does not allow for causal inferences. Because this study uses secondary data with pre-defined variables, there is some data limitation e.g. “perceived distance to health facility as a problem” was used as an independent variable, instead of computed travel time/actual distance to health facility. In addition, the effect of marital status as a determinant of health facility delivery could not be accessed due to multi-collinearity. However, the study remains significant because it uses nationally representative data to determine predictors of health facility delivery among young Nigerian women.

## Conclusion

Higher education attainment, having fewer children, attending frequent antenatal visits, being rich, having a partner with higher than secondary education, living in a community with higher levels of female education, skilled prenatal support, and higher levels of transportation support were associated with delivering at a health facility among young women. An understanding of these predisposing factors can guide maternal health programmes and schemes. These include promoting girl child education, encouraging respectful maternal care, reducing financial barriers in access to healthcare, promoting antenatal care, and improving skilled birth attendants and transportation support in disadvantaged communities should be encouraged.

## Data Availability

Data can be downloaded the measure DHS website https://dhsprogram.com after registration and requesting access.

## Abbreviations

ANC: Antenatal care
AOR: Adjusted odds ratio
CMC: Century month code
DHS: Demographic health survey
EAs: Enumeration areas
FCT: Federal capital territory
HIV: Human Immunodeficiency Virus
ICC: Intra cluster correlation
NDHS: Nigerian demographic and health survey
NHREC: National health research ethics committee of Nigeria
NPHC: The population and housing census of the federal republic of Nigeria
PSU: Primary sampling unit
VIF: Variance Inflation Factor
WHO: World health organization
